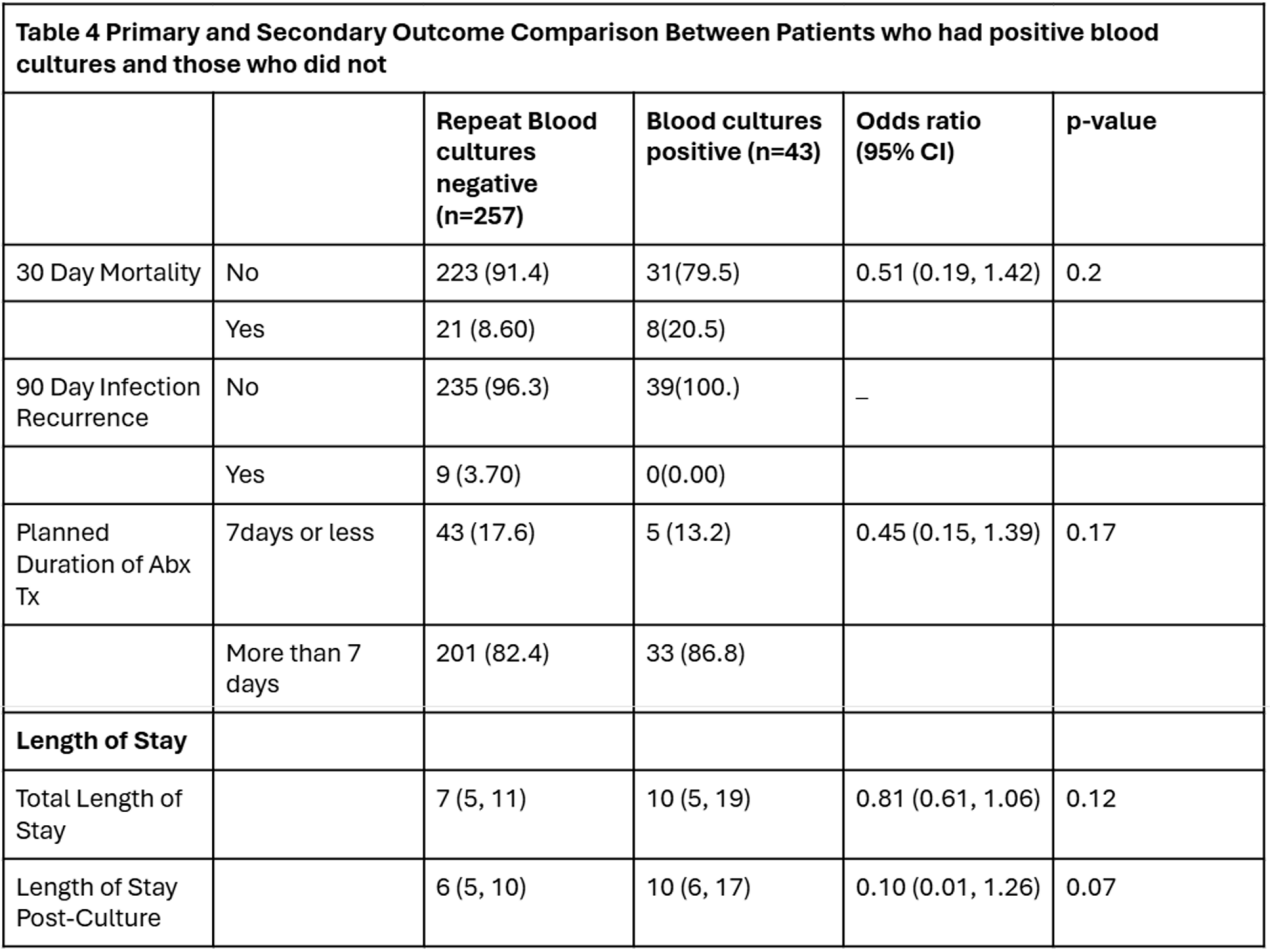# 198 Establishing Infection Prevention and Control Centers of Excellence in Ethiopian Hospitals

**DOI:** 10.1017/ash.2026.10586

**Published:** 2026-06-23

**Authors:** Eric Dai, Thomas Risoleo, James Kuo, Maureen Campion, Kap Sum Foong, Majd Alsoubani

**Affiliations:** 1 Tufts University School of Medicine; 2 Tufts Medical Center; 3 Tufts Medical Center, Tufts University School of Medicine

## Abstract

**Background:** Gram-negative bacteremia (GNB) accounts for a substantial proportion of both community-onset and nosocomial bloodstream infections and carries significant morbidity and mortality. The utility of follow-up blood cultures(FUBC) for GNB remains unclear. Prior studies have demonstrated associations with prolonged hospitalization and antibiotic duration without clear reinfection or mortality benefit. **Methods:** We conducted a retrospective cohort study of adults (<18 years) admitted to a three-hospital healthcare system with GNB from 4/1/2024 to 1/31/2025. Patients who died within 24 hours of the initial positive culture or had hospital stays < 35 days were excluded. Clinical variables including organism, infection source, presence of indwelling catheters or other devices, immunocompromising conditions, severity scores (qSOFA, PITT), as well as intensive care unit admission (ICU) were also collected. Primary outcomes were 30-day mortality and 90-day reinfection. We used chi-square and Fisher’s exact test to compare categorical variables and Mann-U Whitney to compare continuous variables based on repeat blood cultures. We used the univariate screen (p<0.1) and prespecified variables that we thought might impact repeat blood cultures in an inverse probability of treatment-weighted models using propensity scores. **Results:** There were 372 identified eligible patients with GNB. These patients were split based on whether they had FUBC or not. There were no significant differences in immunocompromised status, co-morbidities, or age between the two groups. The most common organisms detected were Enterobacterales, followed by Pseudomonas. The most common source of infection was overwhelmingly genitourinary, followed by gastrointestinal and hepatobiliary. There were no significant differences between those who did and did not undergo FUBC for odds of 30-day mortality (OR 0.74 (0.36-1.55)) or 90- day reinfection (OR 0.86 (0.16-4.47)). However, FUBC were associated with significantly longer median hospital stay (7 days vs 5 days, p < 0.001) and higher odds of receiving <7 days of antibiotics (OR 2.28, (1.23-4.25), p = 0.009). Persistent bacteremia despite appropriate therapy occurred more frequently among patients with ICU admission, though clinical impact could not be fully assessed. Additionally, patients with an Infectious Diseases consult were more likely to have FUBC. No other subgroup was significantly more likely to have blood cultures repeated. **Conclusions:** The use of FUBC in GNB did not demonstrate improvement in mortality or reinfection rates. Instead, FUBC correlated with prolonged hospitalization and extended antibiotic therapy. It remains unclear whether FUBC offers significant clinical benefit in higher-risk groups. Further research is needed to clarify the role of FUBC in critically ill patients.

**Figure:**
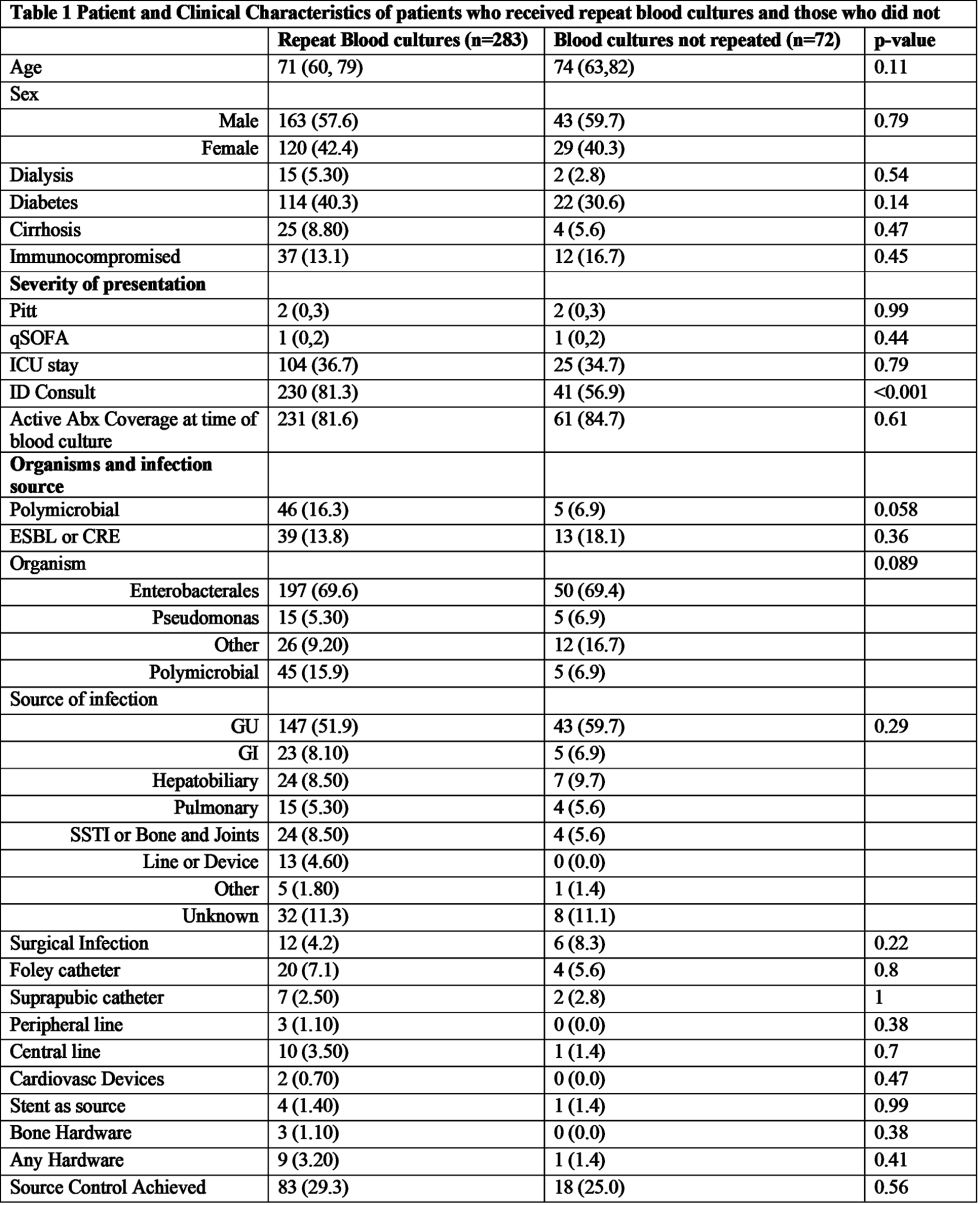

**Figure:**
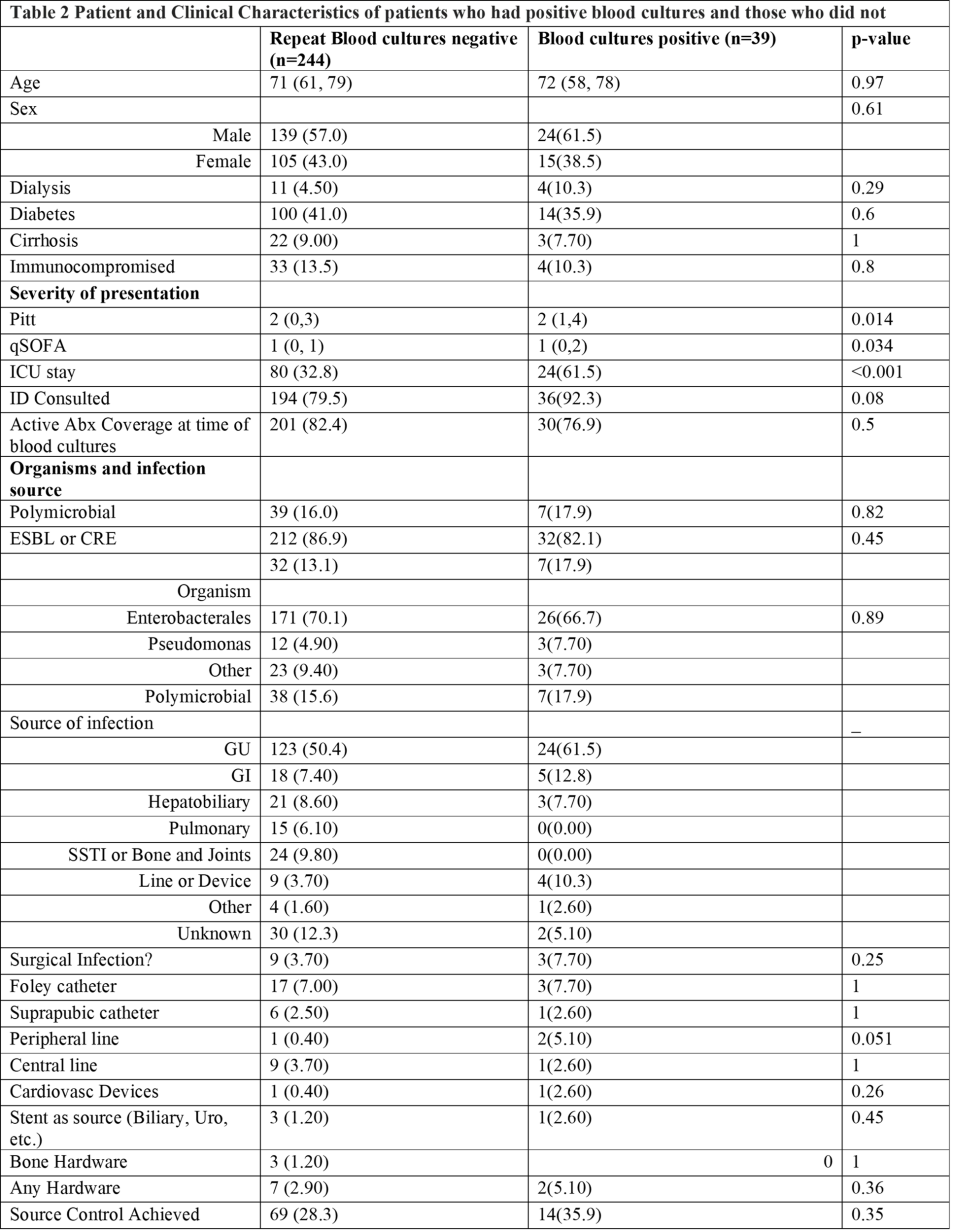

**Figure:**
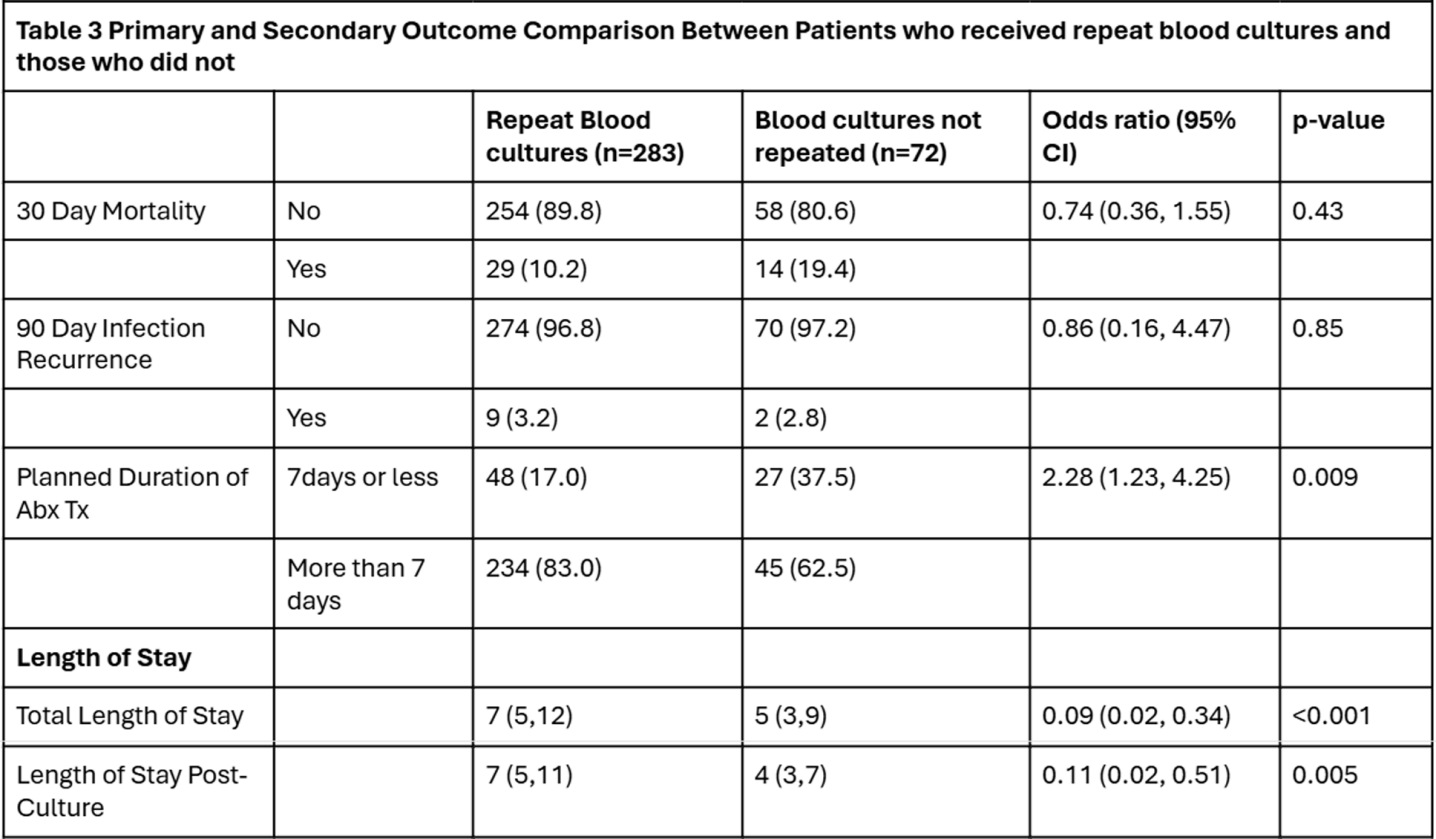

**Figure:**